# The Global Reciprocal Reprogramming between Mycobacteriophage SWU1 and Mycobacterium Reveals the Molecular Strategy of Subversion and Promotion of Phage Infection

**DOI:** 10.3389/fmicb.2016.00041

**Published:** 2016-01-28

**Authors:** Xiangyu Fan, Xiangke Duan, Yan Tong, Qinqin Huang, Mingliang Zhou, Huan Wang, Lanying Zeng, Ry F. Young, Jianping Xie

**Affiliations:** ^1^Institute of Modern Biopharmaceuticals, State Key Laboratory Breeding Base of Eco-Environment and Bio-Resource of the Three Gorges Area, Key Laboratory of Eco-environments in Three Gorges Reservoir Region, Ministry of Education, School of Life Sciences, Southwest UniversityChongqing, China; ^2^Department of Biotechnology, School of Biological Science and Technology, University of JinanJinan, China; ^3^Department of Biochemistry and Biophysics, Center for Phage Technology, Texas A&M UniversityCollege Station, TX, USA

**Keywords:** transcriptome, mycobacteriophage, mycobacterium, interaction, ion channel

## Abstract

Bacteriophages are the viruses of bacteria, which have contributed extensively to our understanding of life and modern biology. The phage-mediated bacterial growth inhibition represents immense untapped source for novel antimicrobials. Insights into the interaction between mycobacteriophage and *Mycobacterium* host will inform better utilizing of mycobacteriophage. In this study, RNA sequencing technology (RNA-seq) was used to explore the global response of *Mycobacterium smegmatis* mc^2^155 at an early phase of infection with mycobacteriophage SWU1, key host metabolic processes of *M. smegmatis* mc^2^155 shut off by SWU1, and the responsible phage proteins. The results of RNA-seq were confirmed by Real-time PCR and functional assay. 1174 genes of *M. smegmatis* mc^2^155 (16.9% of the entire encoding capacity) were differentially regulated by phage infection. These genes belong to six functional categories: (i) signal transduction, (ii) cell energetics, (iii) cell wall biosynthesis, (iv) DNA, RNA, and protein biosynthesis, (v) iron uptake, (vi) central metabolism. The transcription patterns of phage SWU1 were also characterized. This study provided the first global glimpse of the reciprocal reprogramming between the mycobacteriophage and *Mycobacterium* host.

## Introduction

Bacteriophages, the viruses of bacteria with an estimated size of 10^31^ in the biosphere (Whitman et al., 1998), represent an enormous resource for biomedicine and biotechnology, as seen by the growing interest in their therapeutic and food safety applications (García et al., 2008; Monk et al., 2010). Insights into the evolutionary arms race between phage and bacterium have revealed many new molecular machineries such as the widespread bacterial defense system called CRISPR/Cas, which in turn has inspired revolutionary genome editing tools (Barrangou et al., 2007), and exciting novel approaches for antimicrobials discovery (Liu et al., 2004; Samaddar et al., 2016). Well-characterized bacteria hosts,including *Escherichia coli, Salmonella typhimirium*, and *Bacillus subtilis*, have made significant contribution to the understanding of factors essential for phage replication, assembly, and lysis (Young, 2013; Sun et al., 2014).

New methodologies are increasingly used to characterize the interaction between phage and its host, such as the microarrays for profiling phage transcription throughout the T4-*E. coli* infection cycle, and the host during phage infection being profiled (Poranen et al., 2006; Ravantti et al., 2008; Fallico et al., 2011). RNA sequencing (RNA-seq) offers unique opportunity for in-depth interrogating reciprocal reprogramming of phage and bacterium during the phage infection cycle (Garber et al., 2011). However, clinical relevant phage–bacteria interactions are scarce and urgently needed. *Mycobacterium* includes notorious pathogens causing serious diseases in mammals, such as tuberculosis (*Mycobacterium tuberculosis*) and leprosy (*M. leprae*). More than 5850 mycobacteriophages, bacteriophage known to infect mycobacteria, have been isolated using a single host strain, *Mycobacterium smegmatis* mc^2^155, over 600 of which have been completely sequenced (http://phagesdb.org/). Most mycobacteriophages are isolated from America. We have isolated and characterized the first mycobacteriophage SWU1, a lytic phage, from China soil (Fan et al., 2012). In this study, we used RNA-seq and functional assay to characterize the reciprocal reprogramming between SWU1 and *Mycobacterium*, with special aim to unveil how mycobacteriophages usurp the biomolecular machinery of their mycobacterial host.

## Materials and methods

### Bacterial strains, phage, and media

*M. smegmatis* mc^2^155 and mycobacteriophage SWU1 were used for profiling the global gene expression dynamics of the host response to phage infection. *M. smegmatis* mc^2^155 and SWU1 were grown as described previously (Fan et al., 2012; Chen et al., 2013).

### Sample collection for illumina RNA deep sequencing (RNA-seq) technology

An overnight culture was diluted with fresh medium (200 ml) and grown at 37°C until the optical density at 600 nm (OD_600_) up to 2. The culture was harvested by centrifugation, washed twice to remove Tween-80 using fresh 7H9 (without Tween-80), resuspended using MP buffer, and halved (about 10^11^ cells per culture). One portion of the cell culture was infected with SWU1 with a multiplicity of infection of 10 (Ravantti et al., 2008); the other portion was a non-infected control. Cultures were maintained at 37°C. Samples for RNA isolation were taken from infected cultures at time points of 30, 180, and 315 min p.i (named inf_30, inf_180, and inf_315). Sample for RNA isolation was also taken from uninfected cultures at time points of 30 min p.i (named uninf_30). The RNA of inf_30, inf_180, and inf_315 was used to study early, middle, and late transcriptional feature during the SWU1 replicative cycle; The RNA of inf_30, and uninf_30 was used to profile the *M. smegmatis* mc^2^155 response at the early infection by Mycobacteriophage SWU1.

### RNA extraction

For every sample, the cultures were centrifuged at 12,000 r.p.m. for 10 min at 4°C. Cell pellets were snap frozen in liquid nitrogen and stored for subsequent RNA isolation. Total RNA was extracted using Trizol (Liao et al., 2013). The concentration, quality and integrity were determined using a NanoDrop spectrophotometer and an Agilent 2100 Bioanalyzer. The RNA integrity (RIN) value of samples were 6.9 (uninf_30), 8.6 (inf_30), 8.6 (inf_180), and 8.8 (inf_315) (Figure S1).

### cDNA library construction, illumina sequencing, data processing, and analysis

Ribo-Zero™rRNA Removal Kit (Bacteria) was used to remove rRNAs and enrich mRNA. The mRNA was fragmented and used as template to synthesize first-stranded cDNA with reverse transcriptase and random hexamer-primers. Second-stranded cDNA was synthesized using RNase H and DNA polymerase I. These double-stranded cDNA fragments underwent process of end repair, addition of a single “A” base and ligation of adapters (TruSeq™ RNA Sample Prep Kit, Illumina). Adaptor modified fragments were selected by AMPure XP beads and amplified through PCR to create the final cDNA library. Transcriptome sequencing was carried out on an Illumina HiSeqTM2000 platform using 2 × 100 bp reads at the Beijing Novogene company (Beijing, China).

Raw data (raw reads) of fastq format were firstly processed through in-house PERL scripts. In this step, clean data (clean reads) were obtained by removing reads containing adapter, reads containing ploy-*N* (*N* > 10%) and low quality reads from raw data. At the same time, Q20, Q30, and GC content sequence of the clean data were calculated. Based on high quality clean data, all the downstream analyses were carried out.

The high quality reads were mapped to the genome of *M. smegmatis* mc^2^155 and SWU1. For differential expression analysis, the read counts were adjusted by edger program package through one scaling normalized factor (Robinson et al., 2010). Differential expression analysis of two conditions was performed using the DEGSeq R package (1.12.0). The *P*-values were adjusted using the Benjamini and Hochberg method. Corrected *P*-value of 0.005 and log2 (Fold change) of 1 were set as the threshold for significantly differential expression. Different expression genes between infected 30 min and uninfected 30 min can be found as Table S1 online.

KEGG is a database resource for understanding high-level functions and utilities of the biological system (Kanehisa and Goto, 2000), such as the cell, the organism, and the ecosystem, from molecular-level information, especially large-scale molecular datasets generated by genome sequencing and other high-throughput experimental technologies (http://www.genome.jp/kegg/). We used KOBAS software (Xie et al., 2011) to test the statistical enrichment of differential expression genes in KEGG pathways.

### Measurements of ion fluxes

For the ion flux experiments, we harvested a log-phase culture of *M. smegmatis* using centrifugation and washed them with MP buffer to remove Tween-80. This cell suspension was kept on 4°C until used. SWU1 was added into the cell suspension (MOI = 10). The same volume of MP buffer was added as negative control. The concentrations of ions (potassium, sodium, calcium, magnesium, ferrum, manganese, barium, cesium, strontium, aluminum) in the solution were detected by OPTIMA ICP-OES 2100DV (PerkinElmer) in 0 and 30 min using published protocol. Two independent experiments were performed for all measurements (Xiang et al., 2011).

### Validation of transcript levels using real-time PCR

Total RNA samples for real-time PCR were isolated and purified from those samples (uninf_30 and inf_30) by the method described above. Used purified RNA as templates, cDNA production was synthesized using the Transcriptor First Strand cDNA Synthesis Kit (Roche) and was used as templates for real-time PCR. Table S2 lists gene targets and oligonucleotide primers used for Real-time PCR. Real-time PCR was carried out using the SsoAdvanced™ SYBR® Green supermix (BIO-RAD) in Bio-Rad PTC-200 thermal cyclers. 16s rRNA gene was included as control. Three independent experiments were performed.

### Statistical methods

All analyses were carried out by SPSS software (version 13.0 for Windows). To analyze the differences between groups, we used a one-way ANOVA test. A *p-*value of < 0.05 was defined statistically significant.

## Results

### Waves of transcription of mycobacteriophage SWU1 genome

There are a great number of mycobacteriophages sequenced by Graham Hatfulls group (Pope et al., 2015). Using previously reported approach (Hatfull et al., 2006), SWU1 (GenBank accession number: JF946695) belongs to mycobacteriophage A2 Subcluster of Cluster A. The number of Subcluster A2 phages is 41 before 22 December 2015 (http://phagesdb.org). Comparative genomics analysis shows that all Subcluster A2 phages are similar to SWU1 (Figure S2). SWU1 can be a representative of the Subcluster A2 mycobacteriophages.

**Figure 1:**
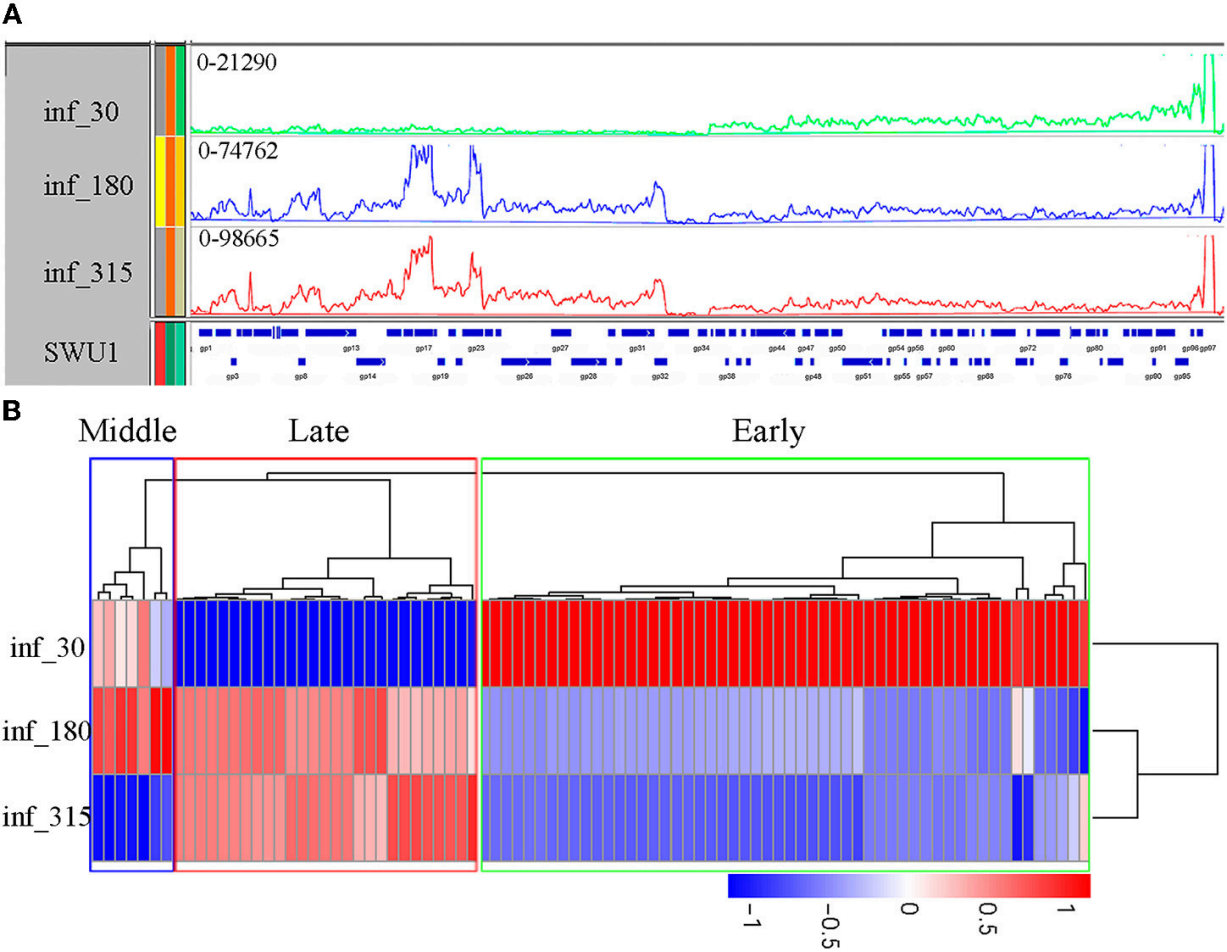
**Transcription of the SWU1 genome determined by RNA-seq**. **(A)** Transcription patterns in the SWU1 genome determined by RNAseq. RNA was isolated during early SWU1 infection (green), middle SWU1 infection (blue), and late SWU1 infection (red). At all time points, one sample was taken for sequencing (Illumina HiSeqTM2000). The Y-axis is the number of RNAseq reads. The X-axis is the map of the SWU1 genome. **(B)** Cluster analysis of differentially expressed genes of SWU1. Based on log_10_ (RPKM+1), we cluster the differentially expressed genes in different phase of SWU1 infection. Red stands for high expressed genes; blue stands for low expressed genes. In early phase of SWU1 infection, a lesser number of genes were transcribed. Most of genes transcribed were located on the right arm of SWU1 genome. In middle and late phase of SWU1 infection, most of genes transcribed were located on the left arm of SWU1 genome.

One-step growth analysis as described in prior report (Fan et al., 2015) revealed that the eclipse period of SWU1 was about 30 min, and burst period about 270 min. To define the gene expression of SWU1 during infection, we used RNA-seq to determine transcription profiles (Figure 1A) in early, middle, and late phage of SWU1 infections, namely 30, 180, and 315 min after absorption, respectively, according to the one-step growth curve. Multiplicity of infection (MOI) of 10 which can infect most hosts (Ravantti et al., 2008), was used in the experiment of SWU1 infection. Clustering of differentially expressed genes (Figure 1B) showed that some highly expressed genes located on the right arm of SWU1 in early phase of SWU1 infection, while other highly expressed genes located on the left arm in middle and late phase. A list of genes displaying three different expression profiles can be found as Table S3 online. Early proteins involved in the bacteriophage–host interaction might be of great value for further study. A promising early protein is SWU1 gp64 that can inhibit the growth of *M. smegmatis* and *E. coli* (our unpublished data).

### Overview of phage-induced host global gene expression alteration

**Figure 2:**
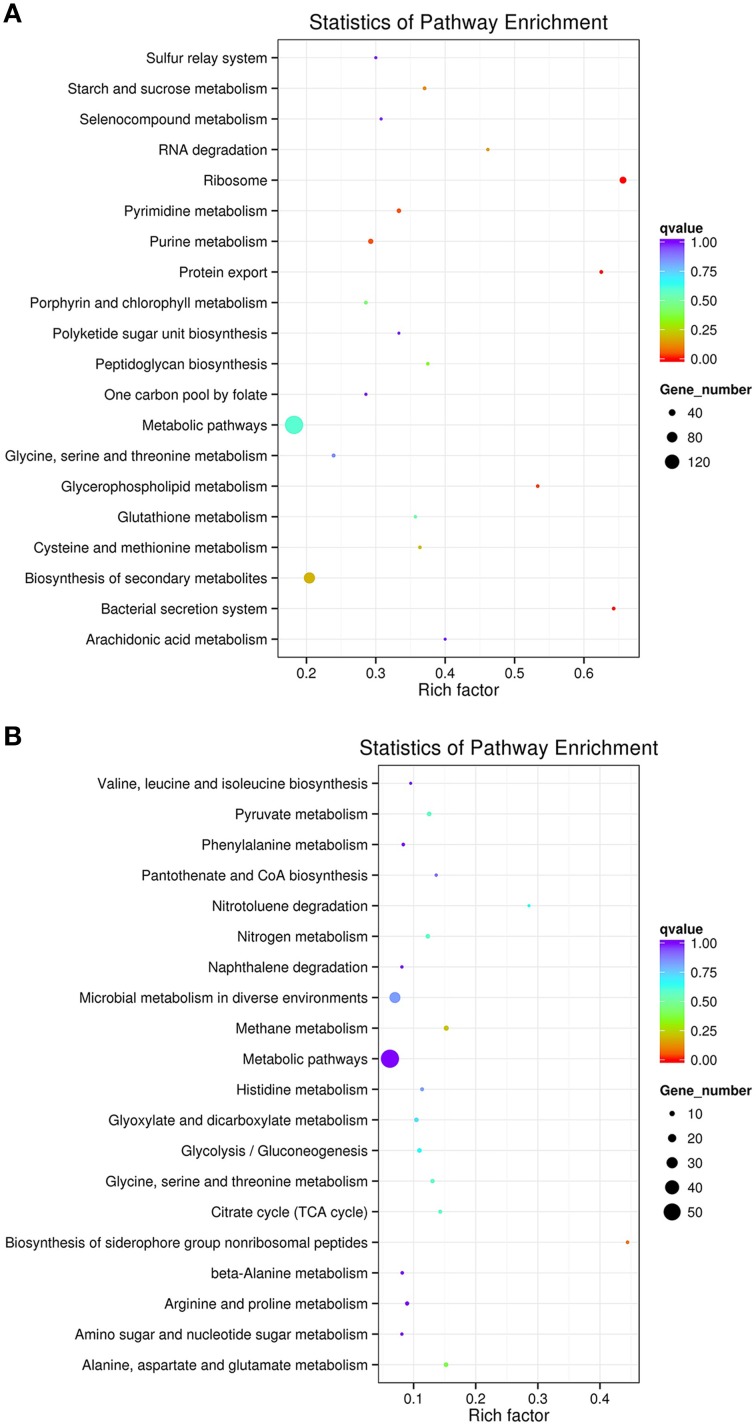
**The scatter plot of KEGG pathways enriched for differential expression genes**. Panel **(A)** shows up-regulated pathways. Panel **(B)** shows down-regulated pathways. The Y-axis is different KEGG pathway. The X-axis is the number of rich factor. The color of circles stands for the *q*-value of pathway. The range of q value is from 0 to 1. The size of circles stands for gene number. Some up-regulated genes associated to ribosome, protein export, bacterial secretion system, glycerophospholipid metabolism, and RNA degradation were enriched. Some down-regulated genes associated to siderophore non-ribosomal peptides and nitrotoluene degradation were enriched.

To profile *M. smegmatis* mc^2^155 gene expression dynamics induced by SWU1 infections, we used RNA-seq to determine the transcriptome of the cell culture infected with SWU1 and the non-infected control culture at 30 min after absorption. The phage SWU1 infection significantly shifted host gene expression. The expression of 1174 (825 up-regulated genes; 349 down-regulated genes) genes was changed at least two-fold, up to 16.9% of *M. smegmatis* mc^2^155 genome encoding capacity. Except 263 function unknown genes, 911 genes can be assigned to different specific pathways using KEGG (Figure 2). Up-regulated pathways include ribosome, protein export, bacterial secretion system, glycerophospholipid metabolism, RNA degradation. Down-regulated pathways include the biosynthesis of siderophore non-ribosomal peptides, nitrotoluene degradation.

### SWU1 infection alters the expression of genes involved in signaling

Signaling is essential to organisms. Mycobacterial serine/threonine protein kinases (STPKs) play important roles in growth, pathogenesis, and cell wall metabolism. During SWU1 infection, some *M. smegmatis* mc^2^155 genes involved in signaling were altered. They are listed in Table S4. Some genes encoded Ser/Thr protein kinases, *pknA, pknB, pknE, pknH*, and *pknF*, were regulated to respond to SWU1 induction (Table S4). *M. smegmatis* mc^2^155 also down-regulated *sigA, sigF*, and the gene encoding anti-sigma F factor antagonist (Table S4). Overexpression of *sigF* resulted in the up-regulation of many cell wall-associated proteins (Williams et al., 2007). However, among the 14 genes directly regulated by SigF, only the homologous genes of Rv3476c, Rv1270c, Rv2400c, and Rv1281c, were up-regulated during phage infection, implicating selective regulation by some unknown phage molecules. In early phase of SWU1 infection, the genes encoding transcription factors had been significant affected. Up to 28 transcriptional factor genes were affected. Among which, 13 were up-regulated; 15 were down-regulated (Table S4). These include WhiA, several FadR-like regulators, Lrp/AsnC, several MerR family regulators, and RpiR. The altered expression of 28 *M. smegmatis* mc^2^155 transcriptional factors showed extensive transcriptional reprogramming in response to SWU1 infection.

### *Mycobacterium* cell energetics and ion fluxes changes in response to phage infection

**Figure 3:**
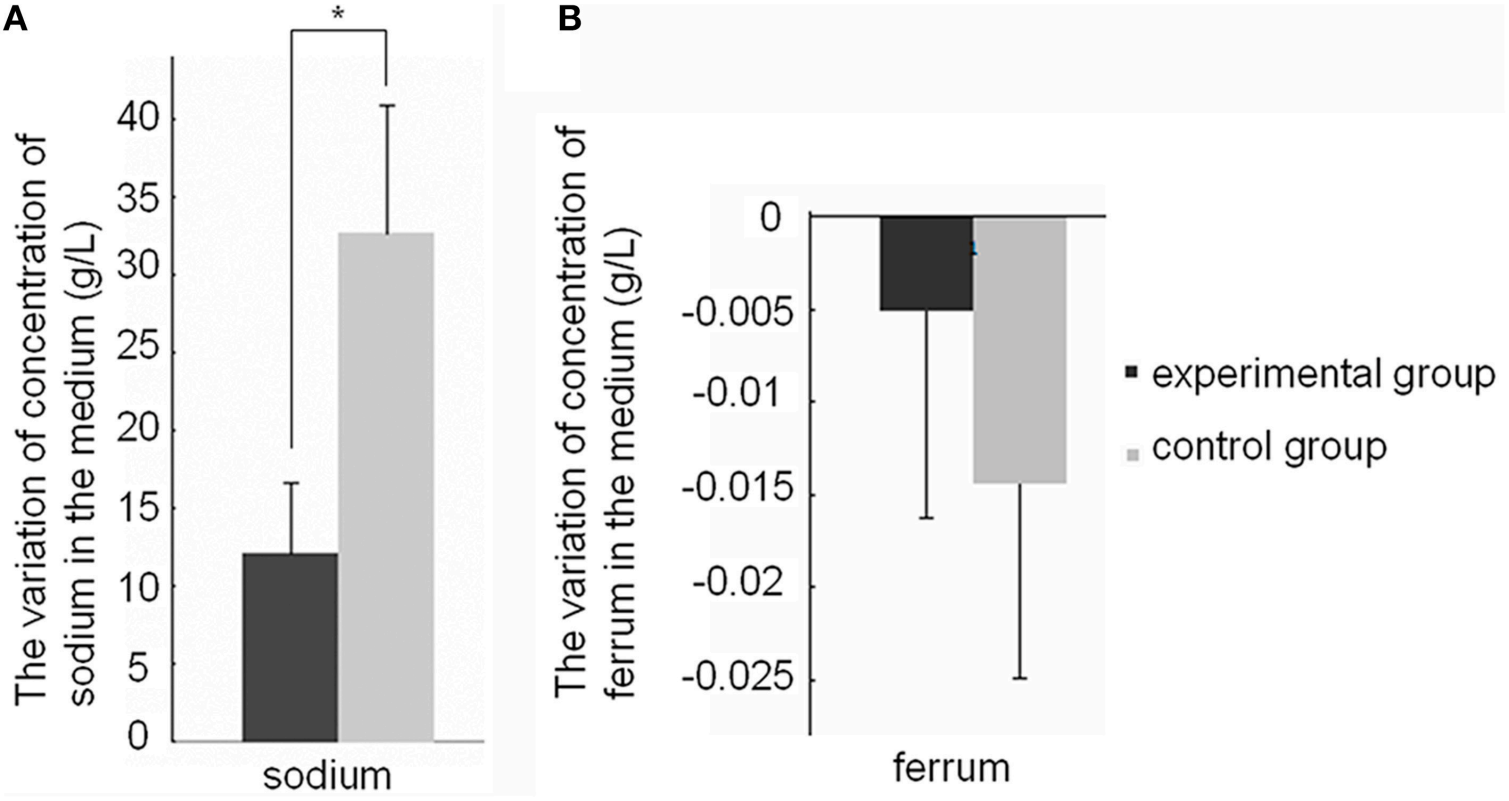
**Effect of the SWU1 infection on sodium and ferrum fluxes**. **(A)** Positive value stands for the concentration of ions increased after SWU1 infected 30 min. **(B)** Negative value stands for the concentration of ions decreased after SWU1 infected 30 min. The capacity of sodium efflux was suppressed after SWU1 infected. Iron uptake system was disrupted by SWU1. The data were averaged from two independent experiments ± s.d. Significant differences (^*^p < 0.05).

In this study, we showed that SWU1 can up-regulate genes involved in the transfer of K^+^ and efflux of K^+^ ions. KdpDE two component system is highly conserved across all bacterial species. Expression of the *kdpD* (encoding sensor kinase) and the *kdpFABC* operon, which encodes the high-affinity K^+^ transporter KdpFABC, was up-regulated early in SWU1 infection. *ftsE* and *ftsX*, encoding FtsEX protein complex, an ABC type transporter proteins, were also up-regulated. KdpD senses intracellular K^+^-limiting signal or extracellular high K^+^ concentration, and KdpE transmits the signal to the *kdpFABC* promoter to regulate the expression of *kdp*FABC (Steyn et al., 2003; Rothenbücher et al., 2006; Laermann et al., 2013). FtsEX protein complex regulates the translocation of K^+^ ion pump proteins KdpA into inner membrane (Ukai et al., 1998). To study the effect of the SWU1 infection on ions fluxes, we measured the concentration level change of some ions (potassium, sodium, calcium, magnesium, ferrum, manganese, barium, cesium, strontium, aluminum) in the medium 30 min after SWU1 infection, using uninfected bacteria as control. Comparing with the control group, the rising values of concentration of sodium decreased significantly (Figure 3A). That meant the capacity of sodium efflux was suppressed after SWU1 infected. In addition, the iron level is significantly reduced. However, the difference was not statistically significant (Figure 3B; Figure S3). For other ions, there was no difference between control group and experimental group (Figure S3). Quite unexpectedly, the increase of the concentration of K^+^ ions in the solution was not significant. The subtle change of the efflux of K^+^ ions beyond the threshold of the measurement might underlie this result.

Transporter proteins are indispensable for the survival of organism. There are 423 transporter proteins in *M. smegmatis* mc^2^155 belonging to five transporter types: ATP-dependent, ion channels, phosphotransferase system (PTS), secondary transporter, and unclassified. Our data showed that after SWU1 infection 119 transporters (28.13%) were regulated (Table S5).

### SWU1 infection induces the expression of the cell wall biosynthetic genes

The expression of the genes involved in the biosynthesis of mycolic acids, arabinogalactan (AG), peptidoglycan (PG), phosphatidylinositol mannosides (PIMs), lipomannan (LM), and lipoarabinomannan (LAM) (Belanger et al., 1996; Crick et al., 2001; Brennan, 2003; Takayama et al., 2005; Jankute et al., 2012; Munshi et al., 2013) had been studied. During SWU1 infection, most genes involved in biogenesis, modification, and regulation of the cell envelope were up-regulated (Table S6).

### SWU1 hijacked *M. smegmatis* mc^2^155 DNA, RNA, and protein synthesis system

Among the 51 *M. smegmatis* mc^2^155 genes related to pyrimidine metabolism, 17 were up-regulated, and one of them was down-regulated during the early infection. Among 82 genes related to purine metabolism, 24 of them were up-regulated, and two of them were down-regulated. The data supported active mobilization of host DNA and RNA synthesis capacity by the phage.

The bacterial transcription largely depends on DNA-directed RNA polymerase. We investigated the components of RNA polymerase holoenzyme, and found that the expression of *rpoA, rpoB, rpoC*, and *rpoZ* was all up-regulated. Moreover, the transcription of *sigA* (*rpoD*) and *sigF* also was up-regulated. The up-regulation of these components demonstrated that SWU1 has usurped the host RNA synthesis machinery.

Ribosome is the factory for protein synthesis. In *M. smegmatis* mc^2^155, there are totally 64 ribosomal proteins, which are the critical components of ribosome. Up to 42 genes in this category were up-regulated. A similar pattern of expression was found for five genes related to aminoacyl-tRNA biosynthesis, which, respectively, involved in the synthesis of L-Aspartyl-tRNA (Asp), L-Glutamyl-tRNA (Glu), L-Glutaminyl-tRNA (Gln), L-Asparaginyl-tRNA (Asn), L-Prolyl-tRNA (Pro), and L-Phenylalanyl-tRNA (Phe). These observations implied that SWU1 also hijacked the bacterial protein expression system to produce virions in the later stage of infection.

### Iron uptake system was disrupted by SWU1

Phage SWU1 infected *M. smegmatis* mc^2^155 down-regulated the genes actively involved in iron homeostasis (*eccC3, mbtF, mbtE, mbtD, mbtB*). *MbtF, mbtE, mbtD*, and *mbtB* are key genes for mycobactin biosynthesis, a class of siderophores for iron uptake in pathogenic and unpathogenic *Mycobacterium* (Snow, 1965). EccC3 is a crucial component of the ESX-3 secretion system important for iron concentration adaptation of *Mycobacterium* (Serafini et al., 2009, 2013). In the absence of Esx-3, *Mycobacteria* failed to use iron-bound mycobactin. The down-regulation of these genes might represent phage tactics to control the bacterial growth.

### The infection of SWU1 activates the metabolism of *Mycobacterium*

*M. tuberculosis* dormancy can benefit the pathogen surviving the stress induced by the accumulation of nitrite under hypoxia within macrophage. The up-regulation of 57 genes and down-regulation of 63 genes were detected in *M. tuberculosis* treated with nitrate (Cunningham-Bussel et al., 2013). These nitrate-regulated genes might underlie the *M. tuberculosis* growth inhibition. Upon SWU1 infection 25 *M. smegmatis* mc^2^155 homologs were differentially expressed (Table S7). Interestingly, 21 genes (84%) showed distinct expression upon the two conditions. This is consistent with previous reports that phages resuscitate and promote growth of dormancy host (Pedulla et al., 2003). The 21 genes might play important roles in regulating the growth of *Mycobacterium*.

### Verification of RNA-seq by real-time PCR analysis

**Figure 4:**
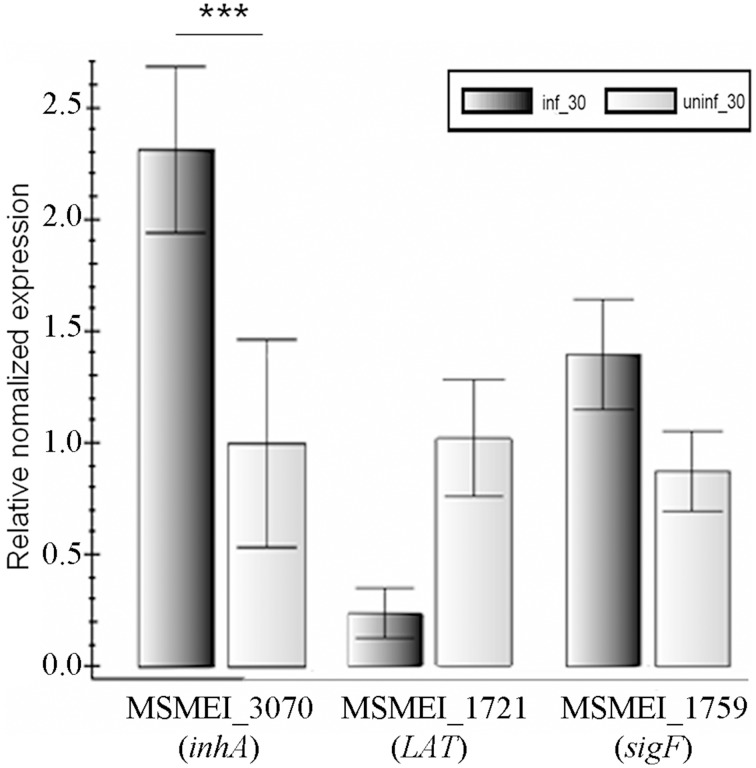
**Verification of RNA-seq results by real-time PCR**. Numbers means the numbering of gene in *M. smegmatis* mc^2^155. The data were averaged from three independent experiments ± s.d. Significant differences (^***^p < 0.001).

Real-time analyses were performed for selected genes to confirm the RNA-seq results. Three genes, namely MSMEI_3070 (*inhA*), MSMEI_1721 (L-lysine-epsilon aminotransferase gene, *LAT*), and MSMEI_1759 (*sigF*) were selected for real-time PCR verification. The three genes were chosen that offered a good representation of the difference in regulation on phage infection. They are involved in cell wall biosynthesis, persistence (metabolism), and signaling, respectively. These genes showed an increase of 5.87 times, decrease of 10.21 times, and increase of 4.62 times, respectively. The real-time PCR results are as following: up-regulated (2.31 times), down-regulated (4.34 times), and up-regulated (1.60 times), which (Figure 4) are in good agreement with the RNA-seq data.

### The shared differentially regulated host genes between SWU1 and other phages

To determine whether there is unifying theme among bacteria–phage interaction, we compared the subset of processes that responded to SWU1 infection with those responding to other phages infection. Genetic response to phage infection has been studied in *Lactococcus lactis* (c2 infection; Fallico et al., 2011), *E. coli* (PRD1 infection) (Poranen et al., 2006), and *Pseudomonas aeruginosa* (PRR1 infection) (Ravantti et al., 2008). We found that some processes are overlap in different phage–host interaction. *L. lactis, E. coli*, and *M. smegmatis* mc^2^155 all regulated stress response genes. Although those stress genes are different, the stress signal transduction is necessary for host response to phage infection. Ribosomal protein encoding genes were significantly up-regulated in *P. aeruginosa* and *M. smegmatis* mc^2^155, which are essential for the production of phage proteins. *L. lactis* and *M. smegmatis* mc^2^155 increases expression of genes involved in cell wall. That suggested that host repaired cell wall to defense phage infection. In addition, *Salmonella typhimurium* (PRD1 infection; (Daugelavicius et al., 1997)), *L. lactis* and *M. smegmatis* mc^2^155 changed cell energetics in response to phage. Manipulating the cell energetics in the bacterial cytosol might represent a phage reproduction strategy. However, the uniqueness of SWU1 might be the control of iron uptake system and the activation the metabolism of dormancy host.

## Discussion

In this paper, we used RNA-seq and functional assay to characterize the reciprocal reprogramming between SWU1 and *Mycobacterium*. Phage SWU1 early transcripts, the key effector proteins which shut off the expression of some host genes, were analyzed. Phage early proteins have been reported to be involved in bacteriophage–host interactions within the infected cell (Rybniker et al., 2008; Roucourt and Lavigne, 2009). The search of candidate effectors altering the host metabolism among the early transcripts of SWU1 will pinpoint the key players in such interaction and inspire future antibiotics or drug target discovery.

To characterize the landscape of host response to phage infection and find the possible key targets phage hijacked during infection, we performed data mining. Significant changes occurred during phage early infection were highlighted. The major targets phage SWU1 hijacked are signal transduction, cell energetics and ions fluxes, cell wall biosynthesis, DNA, RNA, and protein synthesis system, iron uptake system, and some metabolic processes.

During SWU1 infection, *M. smegmatis* mc^2^155 down-regulates some genes about signal transduction. The overexpression of PknA and PknB can hinder the growth of mycobacteria and altered cell morphology (Kang et al., 2005). Phosphorylated substrates of PknA and PknB are involved in diverse cellular events such as the synthesis of polar cell wall, cell shape maintenance, cell division, and peptidoglycan synthesis (Kang et al., 2005; Molle and Kremer, 2010; Prisic et al., 2010). The PknH plays an important role in the LAM/LM (lipoarabinomannan/lipomannan) balance (Molle and Kremer, 2010). PknH can phosphorylate and activate EmbR, the transcriptional repressor of the *embCAB* operon (Sharma et al., 2006). This was consistent with our data which the *embCAB* operon is down-regulated. Decrease of PknF can lead to aberrant septum formation and higher uptake of D-glucose (Deol et al., 2005). SigA is a main sigma factor mediating the enhanced growth of *M. tuberculosis in vivo* (Wu et al., 2004). The high intracellular level of SigF might result from: the expression of *sigF* or the gene of anti-sigma F factor antagonist. SigF is one of the stress response σ factors. *M. smegmatis sigF* can be significant induced under a variety of stress conditions (Singh and Singh, 2008). SigF is also required for the biosynthesis of cell wall in *M. smegmatis*. WhiA could regulate several genes involved in cell division, cell development, and chromosome segregation in *Streptomyces venezuelae* and *B. subtilis* (Bush et al., 2013). FadR-like regulators are involved in amino acid metabolism and some branch point in various metabolic pathways (Vindal et al., 2007). Lrp/AsnC family regulators regulate multiple cellular metabolism globally, such as amino acid metabolism, pili synthesis, DNA metabolism during DNA repair, and recombination (Deng et al., 2011). MerR family regulator is known to be involved in the regulation of gene expression in response to resistance and detoxification (Ramos et al., 2005). The RpiR family of transcriptional regulators can affect virulence determinant synthesis and PPP activity (Zhu et al., 2011). In addition, *dosR* a gene encoding response regulator of a two-component system with two sensor kinases—DosS and DosT, was also up-regulated. The two-component system can respond to nitric oxide, hypoxia, and carbon monoxide stress (Braunstein et al., 2003). IpsA, a LacI-type regulator, which control the biogenesis of cell wall in *Mycobacteria* (Baumgart et al., 2013), was elevated too.

Phages or DNA-containing infectious particles could induce the leakage of K^+^ from the cytosol, a much more complex system than non-specific channel (Boulanger and Letellier, 1992; Daugelavicius et al., 1997). The infection of SWU1 also can up-regulate genes involved in K^+^ ions fluxes. The K^+^ efflux was reported to be associated with an influx of H^+^ and Na^+^ or Li^+^ which compete for entry through the channel. These ion fluxes might underlie the cell depolarization. This selective regulation might underlie a role of host cell energetics in bacteriophage DNA entry. Phage SWU1 infection up-regulated *M. smegmatis* mc^2^155 genes actively involved in the synthesis of F-type ATPase. F-type ATPase can drive an influx of protons (H^+^) through the cell membrane to generate ATP (Fallico et al., 2011). This is consistent with the up-regulation of the biosynthetic genes AsnB. AsnB is a synthetase of asparagines, whose metabolic product is ammonia (Song et al., 2011), a pH buffer. The F-type ATPase-mediated import of protons would result in lower intracellular pH. To maintain the pH homeostasis, *M. smegmatis* mc^2^155 up-regulated the biosynthesis of asparagines, which is the ammonia source for mycobacteria at lower pH *in vitro* (Song et al., 2011). Transient depolarization and repolarization of host membrane was a common effect induced by phage infection (Labedan and Letellier, 1981; Kalasauskaite et al., 1983; Fallico et al., 2011). This might result from the influx or efflux of H^+^ or K^+^. Based on our transcriptome data, we proposed following model. Upon the adsorption of SWU1 to the cell wall of host, the pahge DNA traverses the membranes, an ATP-driven process (Daugelavicius et al., 1997; González-Huici et al., 2004). On this occasion, F-type ATPase is synthesized and H^+^ influxes the cell. The balanced proton motive force is thereby disrupted and the membrane depolarizes. The infected bacteria respond to the signal of depolarization by a repolarization strategy, that opens the potassium channel (Fallico et al., 2011). Subsequently, to resist the influx of H^+^, the infected bacteria induce the biosynthetic genes of asparagines, which leads to ammonia production and is proposed to maintain pH homeostasis. However, evidences are needed to support this largely educated guesswork.

The data of RNA-seq showed that SWU1 disrupted iron uptake system of *M. smegmatis*. However, the experiment (Figure 3B) did not support this hypothesis. We think the major reason is that bacteria have just begun to respond to SWU1 infection from transcription level rather than from translation level after SWU1 infected 30 min. So RNA-seq data revealed that *M. smegmatis* down-regulated the genes involved in iron uptake system. The experiment of ferri concentration measurement showed that the reduction of ferri concentration was not statistically significant.

The infection of SWU1 can up-regulate genes involved in cell wall biosynthesis. As we know, the unique *Mycobacterium* cell wall with three layers represents the first barrier the phage must overcome for successful infection (Brennan, 2003). The cell wall core is the mycolyl arabinogalactan–peptidoglycan (mAGP) complex consisting of peptidoglycan (PG), which is close to the membrane, arabinogalactan (AG) and mycolate. The upper segment of mAGP consists of free lipids, of which cord factor (trehalose 6-dimycolate, TDM) is the most abundant glycolipid and is long held as a virulence factor in the cell wall. The third layer is scattered components, such as, some cell-wall proteins, the phosphatidylinositol mannosides (PIMs), the phthiocerol-containing lipids, lipomannan (LM), and lipoarabinomannan (LAM). Phages must penetrate the host cell wall to deliver their genetic materials. This might mirror that the host is desperate to repair and reinforce the cell wall disrupted by phage to stop the efflux of cytoplasm. Alternatively, the synthesis of the host cell wall might represent a mechanism of superinfection immunity.

Genes predicted to be related to DNA, RNA and protein synthesis system were up-regulated. These data suggested that SWU1 control host bacteria to synthetise DNA, RNA, and protein of phage. Those synthesis systems of *M. smegmatis* mc^2^155 had been hijacked by SWU1. After virus infection, transfer RNA pool of cell will change (Clarkson and Runner, 1971; Kunisawa, 1992). The change is an important guarantee for the reproduction of virus (Wang et al., 2001). We speculated that the up-regulation of those genes related to tRNA synthesis was the survival mechanism of SWU1 for creating favorable environment.

**Figure 5:**
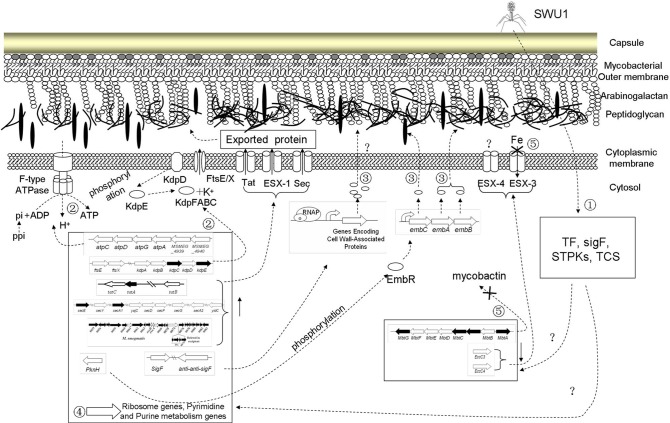
**Putative working model of ***M. smegmatis*** mc^2^155 response to phage SWU1 infection**. Numbers stand for host response to SWU1 infection, which is described in summary. Hollow-out wide arrows stand for those regulated genes. Dotted lines stand for the process of regulatory network. Forks represent that the process is inhibited.

Based on the transcription profiles of both mycobacteriophage SWU1 and *Mycobacterium* during the infection, a usurp strategy employed by the phage and countermeasures mobilized by the host are evident. Phage early transcripts may be the key effector proteins to shut off the expression of some host genes. The major targets phage SWU1 hijacked are DNA, RNA, and protein synthesis system, iron uptake system, and some metabolic processes (Figure 5). Our results also suggested that host regulated some signal transduction system, changed host cell energetics, and inducted the synthetic gene of cell wall to defend the attack of SWU1 (Figure 5). The overall response of *M. smegmatis* mc^2^155 to phage infection involves a complex integrated regulation.

## Author contributions

XF participated in the design of the study, did experiments, analyzed data, and wrote the paper. XD analyzed data and wrote the paper. YT did experiments. QH, MZ, and HW analyzed data. LZ and RY participated in the design of the study and helped to modify the manuscript. JX designed the research and wrote the paper. All authors read and approved the final manuscript.

## Funding

This work was supported by National Natural Science Foundation [grant numbers 81511120001, 81371851, 81071316, 81271882, 81301394], New Century Excellent Talents in Universities [grant number NCET-11-0703], National Megaprojects for Key Infectious Diseases [grant number 2008ZX10003-006], Excellent Ph.D. thesis fellowship of Southwest University [grant numbers kb2010017, ky2011003], the Fundamental Research Funds for the Central Universities [grant numbers XDJK2016D025, XDJK2011D006, XDJK2012D011, XDJK2012D007, XDJK2013D003, XDJK2014D040], Graduate research and innovation project of graduate in Chongqing [grant number CYS14044], The Chongqing Municipal Committee of Education for postgraduates excellence program [grant number YJG123104], The undergraduates teaching reform program [grant number 2013JY201], National Institute of General Medical Sciences of the National Institutes of Health under Award Number R01GM107597, and Shandong Excellent Young Scientist Award Fund [grant number BS2014YY031].

### Conflict of interest statement

The authors declare that the research was conducted in the absence of any commercial or financial relationships that could be construed as a potential conflict of interest.
