# Supplementary material for: The Global Reciprocal Reprogramming between Mycobacteriophage SWU1 and Mycobacterium Reveals the Molecular Strategy of Subversion and Promotion of Phage Infection
Source: Front Microbiol. 2016 Jan 28;7:41. doi: 10.3389/fmicb.2016.00041 (PMC4729954; doi:10.3389/fmicb.2016.00041)
Supplement: Supplementary file 2 [file Table2.DOC]

Table S2. PCR Primers designed for different genes.

| Gene | Forward primers | Reverse primer |
| --- | --- | --- |
| MSMEI_3070 (inhA) | ATCGGGTTCATGCCGCAGAG | GCGACGGTCATCCAGTTGTAG |
| MSMEI_1721 (LAT) | TGGCGTTCGACTGGAAGAGCA | ACCTTGACCGGATCGGTGTTGGT |
| MSMEI_1759 (sigF) | ACCAGATCGACAACCGCGAATCGC | ACGGGAGACGTGCATCTGCGAGAT |
| 16s rRNA gene | GTGAAGCCCTGGACATAAG | CCTTACCTGGGTTTGAC |
