# Supplementary material for: The Global Reciprocal Reprogramming between Mycobacteriophage SWU1 and Mycobacterium Reveals the Molecular Strategy of Subversion and Promotion of Phage Infection
Source: Front Microbiol. 2016 Jan 28;7:41. doi: 10.3389/fmicb.2016.00041 (PMC4729954; doi:10.3389/fmicb.2016.00041)
Supplement: Supplementary file 3 [file Table3.DOC]

Table S3. Transcripts of SWU1 in different phase

| Infection period | The gene of SWU1 |
| --- | --- |
| Early transcripts | gene 97, gene 96, gene 95, gene 94, gene 93, gene 92, gene 91, gene 90, gene 89, gene 88, gene 87, gene 86, gene 85, gene 84, gene 83, gene 82, gene 81, gene 80, gene 79, gene 78, gene 77, gene 76, gene 75, gene 74, gene 73, gene 72, gene 71, gene 70, gene 69, gene 68, gene 67, gene 66, gene 65, gene 64, gene 63, gene 62, gene 61, gene 60, gene 59, gene 58, gene 57, gene 56, gene 55, gene 54, gene 53, gene 52, gene 51, gene 50, gene 49, gene 48, gene 47, gene 46, gene 45, gene 44, gene 43, gene 42, gene 41, gene 40, gene 39, gene 38, gene 37, gene 36, gene 33 |
| Late transcripts | gene 32, gene 31, gene 30, gene 29, gene 28, gene 27, gene 26, gene 25, gene 24, gene 23, gene 22, gene 21, gene 20, gene 19, gene 18, gene 17, gene 16, gene 15, gene14, gene 13, gene 12, gene 11, gene 10, gene 5, gene 4, gene 3, gene 2 |
| Middle transcripts | gene 35, gene 34, gene 6, gene 1, gene 7, gene 8, gene 9 |
