# Supplementary figures and images for: The Global Reciprocal Reprogramming between Mycobacteriophage SWU1 and Mycobacterium Reveals the Molecular Strategy of Subversion and Promotion of Phage Infection
Source: Front Microbiol. 2016 Jan 28;7:41. doi: 10.3389/fmicb.2016.00041 (PMC4729954; doi:10.3389/fmicb.2016.00041)

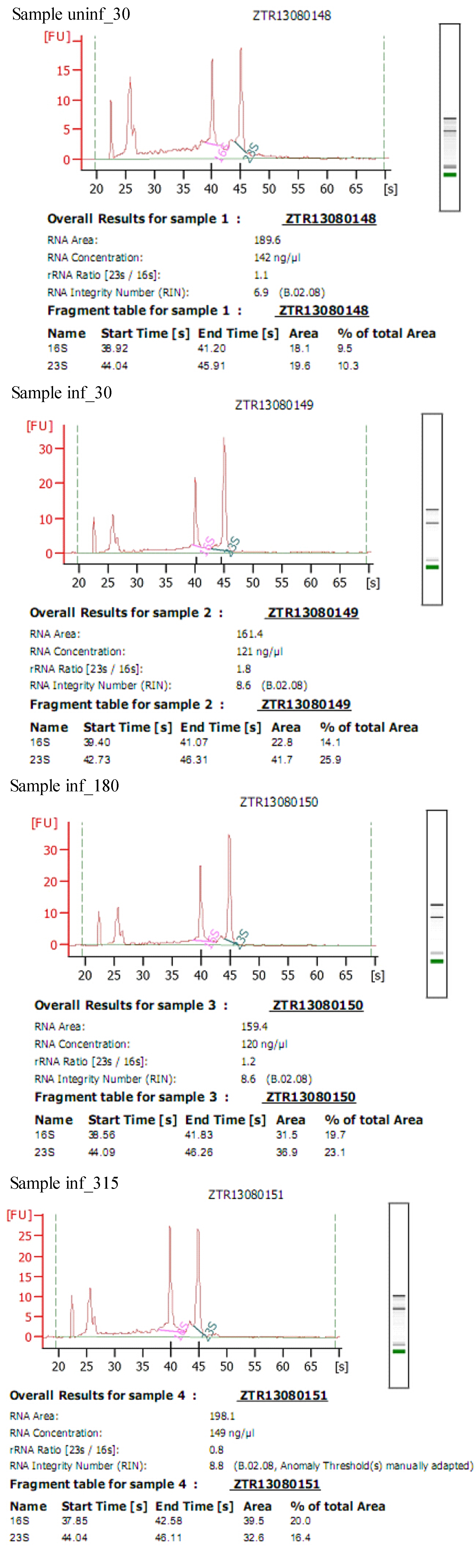

Supplement: Supplementary file 8 [file Image1.TIF]

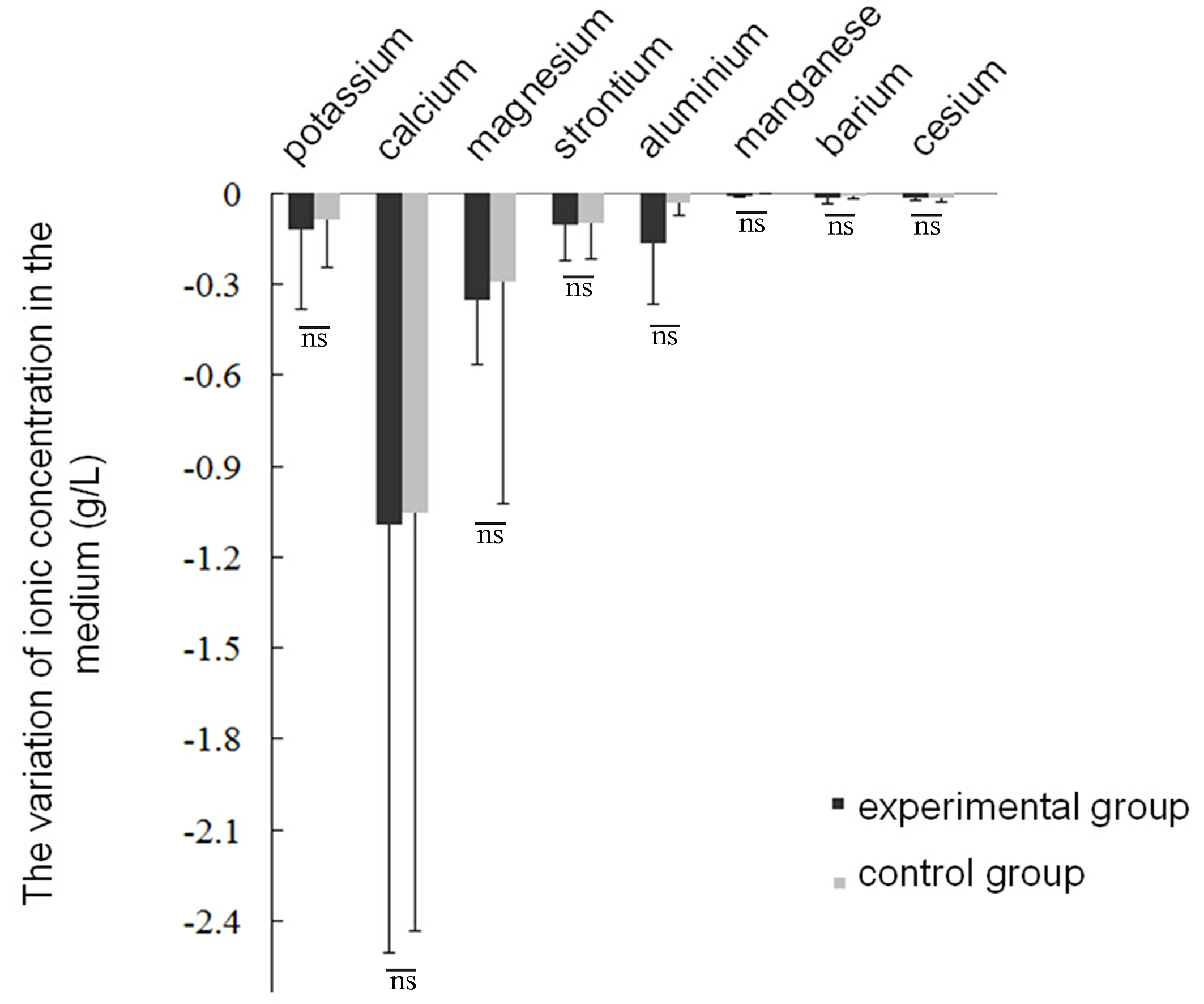

Supplement: Supplementary file 9 [file Image2.TIF]

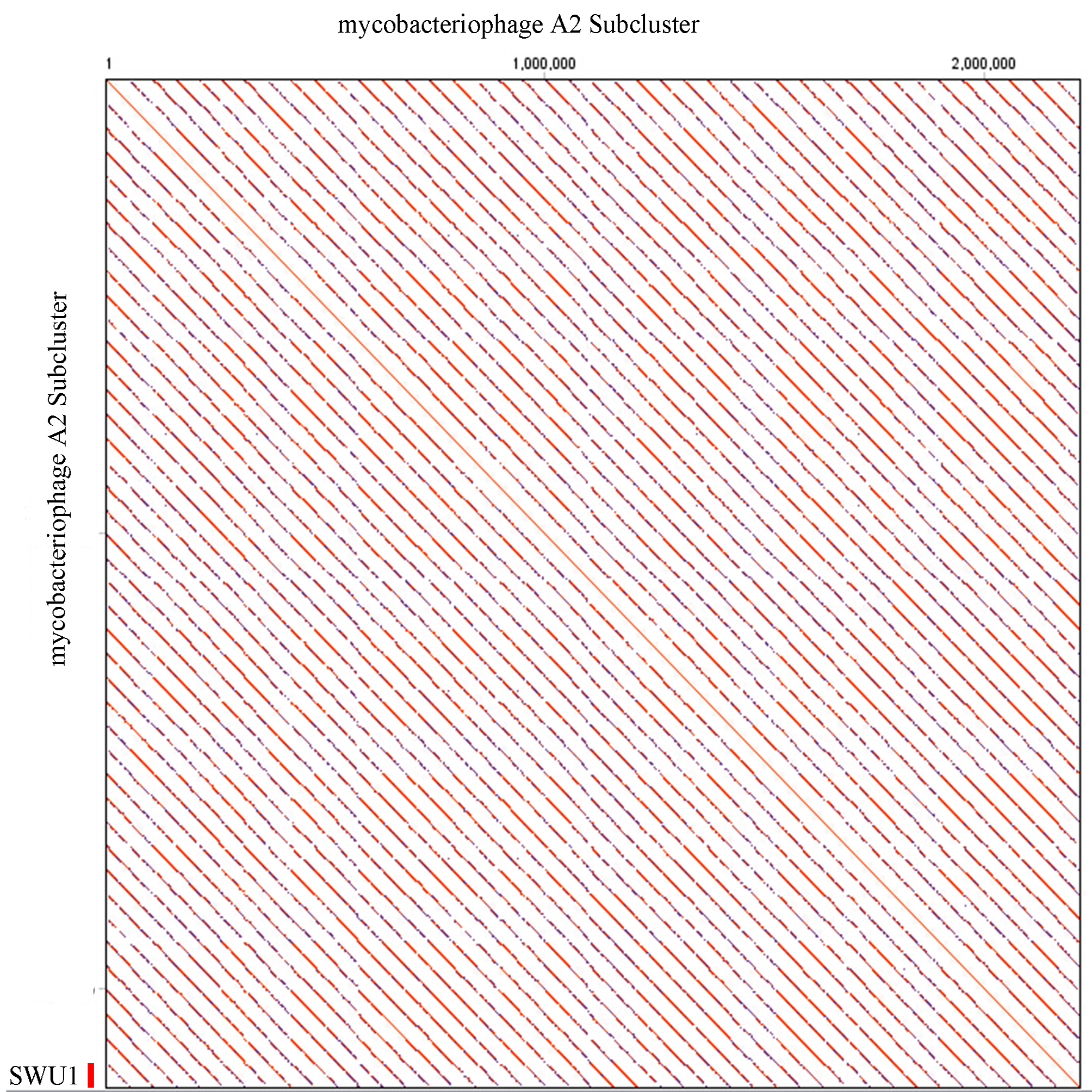

Supplement: Supplementary file 10 [file Image3.TIF]
